# LIVE@Home.Path—innovating the clinical pathway for home-dwelling people with dementia and their caregivers: study protocol for a mixed-method, stepped-wedge, randomized controlled trial

**DOI:** 10.1186/s13063-020-04414-y

**Published:** 2020-06-09

**Authors:** Bettina Sandgathe Husebo, Heather Allore, Wilco Achterberg, Renira Corinne Angeles, Clive Ballard, Frøydis Kristine Bruvik, Stein Erik Fæø, Marie Hidle Gedde, Eirin Hillestad, Frode Fadnes Jacobsen, Øyvind Kirkevold, Egil Kjerstad, Reidun Lisbeth Skeide Kjome, Janne Mannseth, Mala Naik, Rui Nouchi, Nathalie Puaschitz, Rune Samdal, Oscar Tranvåg, Charalampos Tzoulis, Ipsit Vihang Vahia, Maarja Vislapuu, Line Iden Berge

**Affiliations:** 1 Centre for Elderly and Nursing Home Medicine, Department of Global Public Health and Primary Care, Faculty of Medicine, University of Bergen, Bergen, Norway; 2 Department of Nursing Home Medicine, Municipality of Bergen, Bergen, Norway; 3 Department of Internal Medicine, Section of Geriatrics, Yale School of Medicine, New Haven, CT USA; 4 Department of Biostatistics, Yale School of Public Health, New Haven, CT USA; 5 Department of Public Health and Primary Care, Leiden University Medical Center, Leiden, The Netherlands; 6 NORCE Norwegian Research Centre, Bergen, Norway; 7 College of Medicine and Health, University of Exeter, Exeter, UK; 8 Department of Global Public Health and Primary Care, Faculty of Medicine, University of Bergen, Bergen, Norway; 9 Vid Specialized University, Bergen, Norway; 10 Haraldsplass Deaconess Hospital, Bergen, Norway; 11 The Dignity Centre, Bergen, Norway; 12 Centre for Care Research, Western Norway University of Applied Sciences, Bergen, Norway; 13 Norwegian National Advisory Unit on Ageing and Health, Vestfold Hospital Trust, Lillehamner, Norway; 14 Centre of Old Age Psychiatry Research, Innlandet Hospital Trust, Gjøvik, Norway; 15 Department of Health Sciences in Gjøvik, Norwegian University of Science and Technology (NTNU), Gjøvik, Norway; 16 Centre for Pharmacy, Department of Global Public Health and Primary Care, University of Bergen, Bergen, Norway; 17 Department of Cognitive Health Science, Institute of Development, Aging and Cancer, Tohoku University, Tohoku, Japan; 18 Norwegian National Advisory Unit on Women’s Health, Oslo University Hospital, Rikshospitalet, Oslo, Norway; 19 Faculty of Health and Social Sciences, Western Norway University of Applied Sciences, Bergen, Norway; 20 Neuro-SysMed, Department of Neurology, Haukeland University Hospital, Bergen, Norway; 21 Department of Clinical Medicine, University of Bergen, Bergen, Norway; 22 McLean Hospital, Belmont, MA USA; 23 Harvard Medical School, Boston, MA USA; 24 NKS Olaviken Gerontopsychiatric Hospital, Bergen, Norway

**Keywords:** Home-dwelling, Dementia care, Service collaboration, Resource utilization, Caregiver burden, Stepped-wedge randomization, Multicomponent interventions

## Abstract

**Background:**

The global health challenge of dementia is exceptional in size, cost and impact. It is the only top ten cause of death that cannot be prevented, cured or substantially slowed, leaving disease management, caregiver support and service innovation as the main targets for reduction of disease burden. Institutionalization of persons with dementia is common in western countries, despite patients preferring to live longer at home, supported by caregivers. Such complex health challenges warrant multicomponent interventions thoroughly implemented in daily clinical practice. This article describes the rationale, development, feasibility testing and implementation process of the LIVE@Home.Path trial.

**Methods:**

The LIVE@Home.Path trial is a 2-year, multicenter, mixed-method, stepped-wedge randomized controlled trial, aiming to include 315 dyads of home-dwelling people with dementia and their caregivers, recruited from 3 municipalities in Norway. The stepped-wedge randomization implies that all dyads receive the intervention, but the timing is determined by randomization. The control group constitutes the dyads waiting for the intervention. The multicomponent intervention was developed in collaboration with user-representatives, researchers and stakeholders to meet the requirements from the national Dementia Plan 2020. During the 6-month intervention period, the participants will be allocated to a municipal coordinator, the core feature of the intervention, responsible for regular contact with the dyads to facilitate L: Learning, I: Innovation, V: Volunteering and E: Empowerment (LIVE). The primary outcome is resource utilization. This is measured by the Resource Utilization in Dementia (RUD) instrument and the Relative Stress Scale (RSS), reflecting that resource utilization is more than the actual time required for caring but also how burdensome the task is experienced by the caregiver.

**Discussion:**

We expect the implementation of LIVE to lead to a pathway for dementia treatment and care which is cost-effective, compared to treatment as usual, and will support high-quality independent living, at home.

**Trial registration:**

ClinicalTrials.gov: NCT04043364. Registered on 15 March 2019.

## Background

The world’s population is rapidly aging as a result of fewer births and declining mortality rates [1]. The global health challenge of dementia is exceptional in size, cost and impact [2]. According to the World Health Organization, the number of people living with dementia is estimated to be 50 million worldwide, expected to almost triple by 2050 [3]. Despite most people, also from a caregiver perspective, preferring to live longer at home, and to die there, if possible [4, 5], about 30,000 of the estimated 80,000–100,000 persons with dementia (PWDs) in Norway reside in nursing homes [6]. The urbanization of our societies, in particular younger persons moving toward central areas and leaving their older relatives behind, underlines the need for cost-effective service collaboration to provide adequate treatment and care for the aging home-dwelling population.

### Rationale for the present trial

Among the top ten causes of death globally, dementia is the only one that cannot be prevented, cured or substantially slowed [7], leaving disease management, caregiver support and service innovation as the top priority for health policy-makers in the reduction of disease burden. Due to expected positive interactions within the family, interventions supporting them as caregivers not only potentially lessen the caregivers’ burden [8], but could also be beneficial for the PWD (e.g. reducing neuropsychiatric symptoms and delaying nursing home admission) [9, 10]. As such, interventions supporting caregivers hold the potential for better overall resource allocation and utilization [11].

Caring for a PWD comes at a high cost, both individually and at societal level. Caregivers to PWDs have lower perceived health and higher rates of mortality relative to their noncaregiver counterparts [12]. The effect of practical assistance and psychoeducational programs have been evaluated, but most single initiatives have fallen short in reducing the caregivers’ burden [13]. The Maximizing Independence (MIND) at HOME study undertaken in Baltimore, USA, during 2008–2010 included approximately 300 home-dwelling persons with cognitive impairment or dementia in a parallel randomized multicomponent trial [14, 15]. This study showed that 18 months of care coordination through individualized care planning, implementation of a care plan, monitoring and reassessment had beneficial effects on the time to transition from home, number of dementia-related unmet needs, quality of life (QoL) and, importantly, a potentially clinically relevant reduction in self-reported number of hours spent on caregiving tasks, as a measure of caregiver burden [14, 15]. Developing this model further, the MIND at Home-Plus study included an additional 340 persons to evaluate the effect on long-term care placement, hospitalization and health-care expenditures of a 24-month homecare coordination program for PWD [16]. The MIND at Home-Streamlined trial is now refining the intervention to investigate its impact on time to long-term care placement, needs, burdens and QoL in PWDs and their caregivers, as well as cost utilization [17]. Results of the latter study are highly anticipated due to the potential for effective system-level approaches to dementia care [17]. Yet, due to fairly large regional and cultural differences in care organization, there is a need for implementation studies in other countries to explore the generalizability of the program.

A multicomponent intervention is not merely a discrete package of separate components, but a process of changing what complex systems do [18]. Intervening within a complex system involves disrupting prior ways of working while introducing new ones [19]. The degree of complexity can be understood as a relative construct, defined by the number of components, diversity of the intended outcome, number of targeted organizational levels and level of skill required to deliver the intervention [20], while additionally considering the interplay between context, setting and the implementation process [21]. In the COSMOS trial, a randomized implementation hybrid trial carried out in Norwegian nursing homes during 2014–2015, our group successfully developed, implemented and effect evaluated a multicomponent intervention addressing COmmunication, Systematic assessment and treatment of pain, Medication review, Organization of activities and Safety [22]. Overall, the intervention resulted in improved QoL and activities of daily living (ADL), in addition to a decrease in neuropsychiatric symptoms such as agitation and depression as well as a reduction in the number of medications used among nursing home residents [23–27].

To provide cost-effective care while securing the needs of PWDs and caregivers represents a complex health challenge warranting multicomponent interventions implemented in daily clinical practice. Aiming at system-level change, such interventions require stakeholder involvement as well as collaboration within and between different levels of primary and specialist health-care services, nongovernmental institutions, users and researchers, addressing the need for appropriate and coordinated cross-sector action.

### Aim of the LIVE@Home.Path trial

The LIVE@Home.Path trial aims to develop, adapt, implement and effect-evaluate a multicomponent intervention for home-dwelling dyads of PWDs and their caregivers, aiding them to stay safer, longer and more independently at home with cost-effectiveness. In this study, caregivers are defined as family or close friends, equaling informal caregivers. LIVE@Home.Path is an acronym referring to each component of the complex intervention: Learning, Innovation, Volunteer support and Empowerment—At Home Pathway. The primary outcome is resource utilization. This is measured by the Resource Utilization in Dementia (RUD) instrument and the Relative Stress Scale (RSS), reflecting that resource utilization is more than the actual time required for caregiving tasks, but also how burdensome the task is experienced by the caregiver. Importantly, the caregiver burden is individual, and may be related to economic hardship, anxiety, depression, hopelessness, impaired QoL or lack of sleep and time for recreation. This individual perspective underlines the significance of user involvement, reflected in the trial’s slogan: what matters to you? Secondary outcomes include neuropsychiatric symptoms, number of adverse events, use of assistive technology, involvement of volunteers, QoL and clinical global impression of change for the PWD as well as caregivers’ depression, QoL and work performance.

### Main hypothesis

The LIVE intervention will reduce time and resources that caregivers spend in organizing and supporting PWDs’ daily activities, thereby reducing the caregiver burden.

## Methods and design

The LIVE@Home.Path trial is a 2-year, multicenter, mixed-method, stepped-wedge randomized controlled trial (RCT). We aim to recruit 315 dyads of home-dwelling PWDs and their caregivers from the municipalities of Bergen, Bærum and Kristiansand.

### Method of intervention development, implementation and evaluation

#### Development of the LIVE intervention

Based on experiences with two pre-projects— Research Council of Norway sponsor code 261626 (UiB) and 261605 (Haraldsplass Deaconess Hospital)— the intervention was developed in collaboration with user-representatives, stakeholders and scientific partners from the Scientific Advisory Board. To meet the requirements from the Dementia Plan 2020 by the Ministry of Health and Care Services [28], we identified the “big issues” expected to facilitate support for home-dwelling PWDs and their caregivers. As such, we combined and adapted existing knowledge rather than designing new components, contributing to service innovation in the health-care systems. The process was tailored to meet the standards of “Development–evaluation–implementation”, an internationally agreed approach for complex interventions launched by the UK Medical Research Council [29].

#### The LIVE intervention

**Fig. 1:**
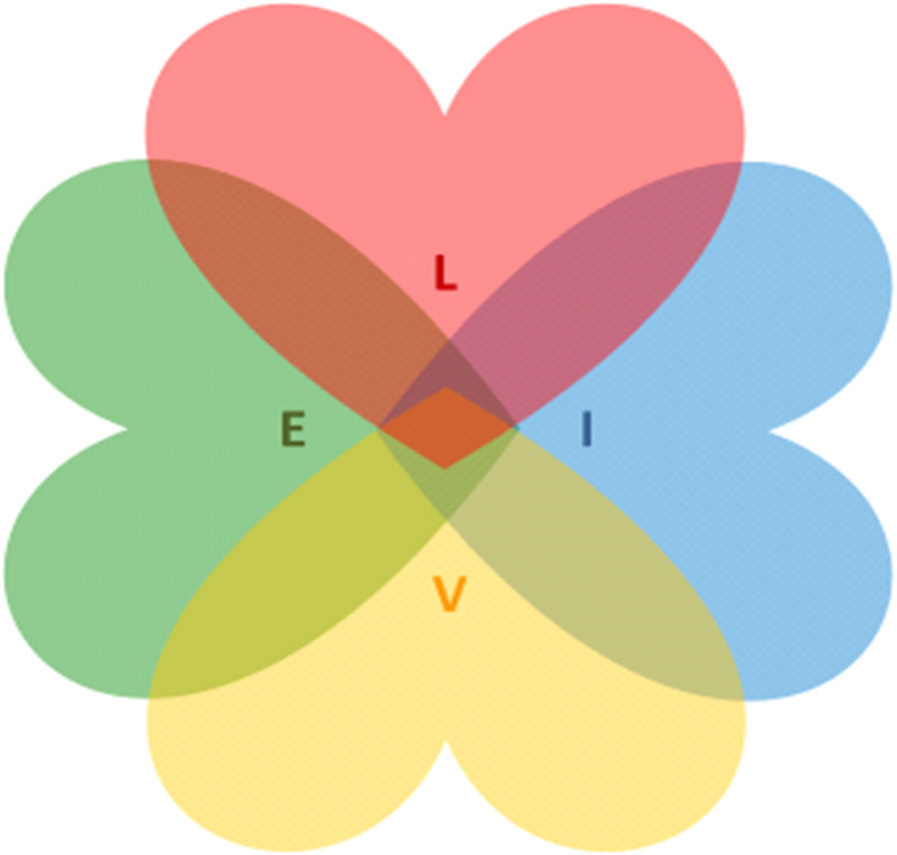
Learning (L), Innovation (I), Volunteering (V) and Empowerment (E)

At the start of the 6-month intervention period, the dyads will be allocated to a municipal coordinator, offering regular contact to assist in finding a pathway throughout the administrative trajectory of dementia care. The coordinator should hold a bachelor degree in health-related science (e.g. nursing, ergo or physiotherapy), and will make a minimum of two home visits, one immediately after the intervention start and the second after approximately 3 months. Supplementary visits will be offered if needed, in addition to monthly telephone calls. During the intervention, the coordinator will introduce the dyads to the different stages of the LIVE intervention: Learning, Innovation, Volunteer support and Empowerment (Table 1, Fig. 1). All components will be carefully adapted to local conditions.

**Table 1:** Components of the LIVE intervention in the LIVE@Home.Path trial

	Learning	Innovation	Volunteer	Empowerment
Content	Local learning programs covering key aspects of the dementia syndrome, coping in daily life, legal, safety and economic issues	Tailored use of welfare technology such as technical aids, cognitive intervention devices and assisted living systems/smart house systems Service innovation Data collection	PWDs often experience social deprivation, and volunteer support is a politically highly prioritized area in Norway Support of relatives	End-of-life care and advance care planning: a repeating process of communication to investigate values and wishes for domestic and institutionalized treatment and care (i.e. “What matters to you?”) Systematic medication review by the PWD’s general practitioner
Participants	PWD Caregivers Coordinators Volunteers Teachers in the municipal and specialized health-care services	PWD Caregivers Coordinators	PWD Caregivers Coordinators Volunteers from nonprofit organizations (The Red Cross, Norwegian Association for Public Health) Volunteer managers	PWD Caregivers Coordinators General practitioner
Actions	Coordinator: • Inform about potential lessons/courses for both PWD and caregiver • Search for practical solutions to ensure participation	Coordinator: • Assess and evaluate usefulness of devices already in use • Inform about additional available welfare technology in the municipality • Inform about specific communication platforms (Jodacare©, Friskus©), social media forums (Facebook©) and applications for tablets (Alight©)	Coordinator: • Inform about volunteer services Volunteer manager: • Match PWD with volunteer after assessment of preferences and wishes	Coordinator: • Initiate systematic medication review with general practitioner • Initiate advanced care planning with general practitioner, including issues on formal next of kin, guardianship • Facilitate application process for home-based services

*PWD* person with dementia

##### Learning

A fruitful learning process is characterized by relevance, timing, confidentiality and reflection as well as fulfilment of expectations regarding content. The Dementia Plan 2020 [28] underlines increased knowledge at all societal levels as crucial for improvements in dementia care. A meta-analysis on the effectiveness of educational interventions supporting caregivers of community-dwelling PWDs found a moderate impact on the caregiver burden, a small effect on depression, but no effect on transition to long-term care [30]. A Norwegian multicenter randomized controlled trial found no reduction in depressive symptoms for PWDs and caregivers after a 12-month psychosocial support program including formal education seminars [31]. Yet coping had a positive impact on the caregiver burden in the latter study, possibly reflecting improved understanding of the caregiver situation [31].

*In practice in the LIVE@Home.Path*: the coordinator will encourage and facilitate that both the PWD and the caregiver participate in local educational programs arranged by the municipality or the specialist health services several times yearly. As an example, the nationally established educational program for relatives of PWDs is developed by the Norwegian Advisory Unit on Ageing and Health [32], and implemented for use in Bergen, Bærum and Kristiansand.

##### Innovation

Innovation is understood as the application of better and more original solutions to meet new requirements, unarticulated needs or existing market needs, or employing established solutions in new areas, both technological, such as information and communication technology (ICT), and organizational. Crucially, the process will result in more effective products, processes, services, technologies or business models being made available for all, including markets, government and society [33]. As such, the LIVE@Home.Path can be viewed as a service innovation, aiming at the development of a clinical pathway for dementia care.

ICT approaches in elderly care are broadly categorized as technical aids, cognitive intervention devices, and sensor and assistive living systems [34]. ICT in dementia care holds potential for optimizing safety at home, reducing caregiver burden and, although the findings are not conclusive [35], possibly also improving cost-effectiveness. Yet we have limited knowledge about which type of devices are used, regarded as useful and requested by caregivers and PWDs at different stages of dementia [36]. Most important, this field requires a careful, individual risk–benefit assessment, as ICT might negatively impact autonomy and privacy, and provide a false sense of safety.

*In practice in the LIVE@Home.Path*: the coordinator will assess and evaluate the usefulness of ICT solutions already in use for PWDs and caregivers and inform about additional relevant welfare technology available in the municipality. The participants will receive information about a newly launched online communication platform tailored to meet the needs of families organizing dementia care (Jodacare©) [37], and be informed about a web page with scheduled activities of relevance (Friskus©) [38]. In Bergen, the participants will be invited to test the prototype Alight©, an application for tablets providing a “digital memory book” developed by Soundio AS and NKS Olaviken Gerontopsychiatric Hospital [39]. Additionally, up to ten participants in Bergen will be invited to test a prototype of the adapted communication platform in collaboration with the Western Norway University of Applied Sciences. Underlining the aspects of service innovation, all data will be collected on tablets owned by the project group via the software SurveyJS [40]. The LIVE@Home.Path trial was selected as a pilot for the development and evaluation of this software, providing secure data transfer and storage on the SAFE server at the University of Bergen for research project with sensitive data. After approval from the principal investigator, researchers affiliated with the project will be given access to the server, avoiding export of data and maintaining high levels of security [41].

##### Volunteer support

Volunteer support is understood as any activities that involves someone spending time, unpaid and of one’s own will, doing something that aims to benefit someone else outside their own families and households [42]. Being important suppliers of unpaid support, it is estimated that volunteers contributed 142,000 full-time equivalents (FTEs) in Norway in 2017 [43]. However, the majority are engaged in sports and culture, and representation in the elderly care sector is sparse [44]. Volunteering among older adults reduces their depressive symptoms, improves self-reported health and functional performance, and increases survival [42, 45]. The volunteers additionally report better health through their own engagement [46, 47]. Volunteerism has contributed to the development of the Norwegian welfare system through identifying and providing solutions to societal challenges [48], being formally integrated into core strategic plans in the health-care sector and being launched as a prioritized political strategy in elderly and dementia care in Norway [49]. Yet we have sparse knowledge about volunteer support schemes for home-dwelling PWDs. To provide better services, understanding of the dynamics, motivations and interactions in volunteerism in dementia care is required.

*In practice in the LIVE@Home.Path*: the coordinator will investigate PWD and caregiver attitudes toward volunteer support, and inform about volunteer services. If this is of interest, the coordinator will contact local volunteer coordinators for nonprofit organizations (The Red Cross [50] and The Norwegian Association for Public Health [51]), aiming at the best possible match of volunteers based on assessment of preferences and wishes.

##### Empowerment

Empowerment in dementia care can be defined as “a confidence building process whereby PWD are respected, have a voice and are heard, are involved in making decisions about their lives and have the opportunity to create change through access to appropriate resources” [52]. The process of advanced care planning (ACP) can increase empowerment for PWDs and their caregivers [26, 27], underlined by the Norwegian policy guidance by the Directorate of Health on diagnosis, treatment and care for PWDs [53]. PWDs do not necessarily die from dementia, they die with it, and the life expectancy after onset of symptoms ranges from 4 to 11 years, depending on age and the presence of comorbidities [54]. The continuing process of communication should be initiated as early as possible in collaboration with the general practitioner as a comprehensive medical examination including revision of medications, enabling the PWD to clarify individual values and wishes for domestic and institutionalized treatment and care (i.e. “What matters to you?”).

*In practice in the LIVE@Home.Path*: the coordinator will schedule a minimum of one appointment at the general practitioner’s office for empowering ACP, including the issues of formal next of kin and guardianship. In addition, a systematic medication review will be undertaken to ensure use of medications in line with diagnoses and symptoms, utilizing recommended guidelines [25].

#### The feasibility study

To evaluate the feasibility and the implementation strategy of the coordinators of the LIVE intervention, a feasibility study was conducted during 2018–2019. Sixteen dyads in Bergen were assigned a coordinator for 6 months, participating in a minimum of two home visits and providing monthly follow up by telephone. One dyad dropped out after a few weeks of participation due to permanent placement in a nursing home, leaving 15 dyads followed by 2 coordinators for assessment. Qualitative individual and focus group interviews utilizing a hermeneutic approach were performed with six dyads, three caregivers and the two coordinators as well as the coordinators’ leader, exploring the usefulness of the coordinator function. This process revealed that the core feature of the coordinator was to support the caregivers in finding, applying and organizing support, and to provide emotional care, support and guidance. The objective of empowering the PWD in the decision-making processes was nonetheless particularly difficult to achieve. This finding was further incorporated into the LIVE intervention for the stepped-wedge RCT, with increased focus on the ACP process and follow up of the GP [55].

#### Implementation process of the LIVE intervention

Implementation research is defined as the scientific investigation concerning the act of carrying an intervention into effect in the real-world setting [56, 57]. Even a superbly designed intervention will fail to change practice if the process of implementation is futile. In the LIVE@Home.Path trial, the implementation can be viewed as a two-stage process: first, from the research team to the coordinators; and, second, from the coordinators to the dyads. The first part encompasses all activities arranged by the research team empowering the coordinators to standardize the implementation of the intervention, such as seminars, development of written material and follow-up of coordinators during the intervention period. Six months prior to the intervention start, kick-off workshops for all involved collaborators in the municipalities, including coordinators and affiliated specialized health services, will be arranged at all study sites, facilitating enthusiasm, collaboration and recruitment of participants. Two weeks before the intervention start, a 2-day implementation seminar for the coordinators will be delivered by the research team at all study sites, training the coordinators through lectures, role-play and discussions (see Additional file 1). Halfway through the 6-month intervention period, a 1-day midway evaluation workshop for the coordinators will be arranged, allowing for discussion of obstacles and pitfalls, which acts as a source for facilitating a more effective and standardized implementation. As a part of the intervention, the research team will contact each coordinator by telephone every 14 days to keep track of the process, discuss potential challenges and follow-up use of the *Checklist for implementation of the intervention*. This ten-page pocket manual will contain a simplified how-to-do description of the intervention components. It will be filled out for each dyad by the coordinator, registering time use and whether each of the distinct LIVE components has been addressed during the intervention period. Additionally, a 30-page tutorial will be developed as a comprehensible introduction to the rationale, method and practical aspects of the conduction of the trial, aimed for an audience not skilled in the research method.

The second part of the implementation process encompasses the coordinator–dyad relationship. The coordinators are obliged to arrange a minimum of two home visits during the intervention period, and provide monthly contact by telephone. The *Checklist for implementation of the intervention* will be used at every contact, and collected by the research team at the end of the intervention, providing documentation for the implementation process.

#### Evaluation of the implementation process

In addition to the midway evaluation, a LIVE conference will be organized for all coordinators at the end of the third intervention period, collecting data on their experiences of the suitability of the single components and the implementation process. Additionally, at data collection after the intervention period, the participants will be asked if and to what extent they were offered the LIVE components, and how often they were contacted by their coordinator. As such, if the LIVE intervention fails to prove an effect on resource utilization, it will be possible to examine whether this is a result of the LIVE components not being tailored to produce such an effect (i.e. that our main hypothesis was wrong) or whether it was caused by a lack of proper implementation. Evaluation of the implementation process will further be investigated by conducting qualitative interviews with the coordinators as part of the mixed-method design.

### Sample size calculations, settings and target populations

The required sample size was calculated to detect a difference of 7 h/week for the primary outcome RUD. Based on the literature, we assumed that the mean number of hours of informal care is 46 h/week with a standard deviation (SD) of 20 h/week [58]. With 80% power and a significance level of 5%, the required sample size was estimated to be 260 dyads. To allow for 20% loss to follow-up, a total of 315 dyads, equaling 105 per municipality, must be included.

Participants will be recruited from memory clinics at local hospitals, from municipal memory teams and after advertisements in general media such as newspapers, radio and TV in Bergen, Bærum and Kristiansand. Bergen is the second largest municipality of Norway with approximately 280,000 inhabitants in 2018, Bærum is ranked the fifth largest with 127,000 inhabitants, while the 92,000 inhabitants of Kristiansand constitute Norway’s sixth largest municipality [59].

PWDs are eligible for inclusion if they: are aged ≥ 65 years; are home-dwelling; have a minimum 1 h/week regular face-to-face contact with the caregiver; are diagnosed with dementia according to standardized protocol [60]; have Mini-Mental State Examination (MMSE) score of 15–25; have a Functional Assessment Staging Test (FAST) score of 4–7; and provide written informed consent. Exclusion criteria are: participation in another ongoing intervention trial; or expected survival < 4 weeks. PWDs are eligible for inclusion regardless of etiology of the dementia and presence of other disorders. Caregivers are eligible for inclusion if they have a minimum of 1 h/week regular face-to-face contact with the PMD and provide written informed consent. As such, both the PWD and the caregiver will be included in the trial, representing a dyad.

### The mixed-method, stepped-wedge randomized control design

**Fig. 2:**
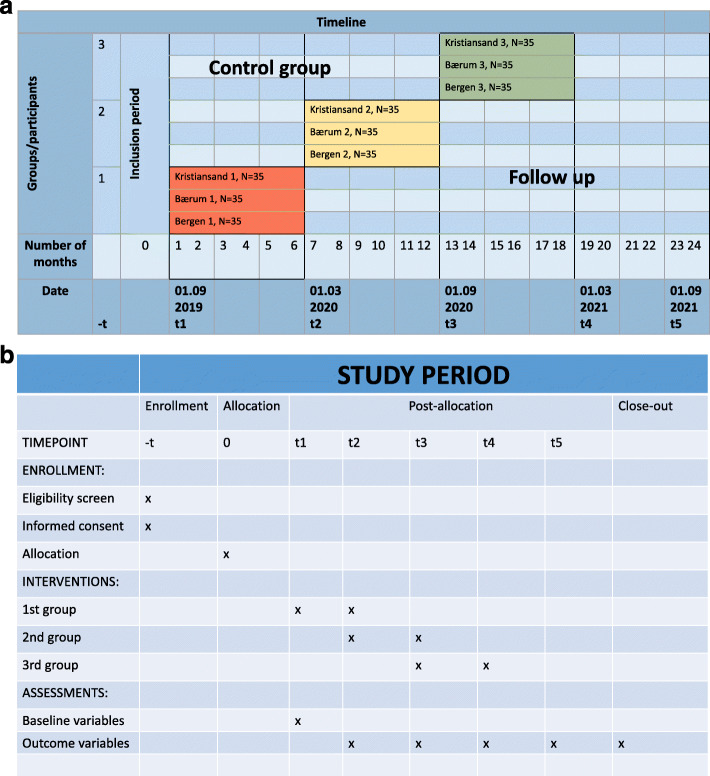
**a** Stepped-wedge randomized control design. The randomization in time takes place at month 0. First group (red) is in the intervention period from month 1 to 6, second group (yellow) from month 7 to 12 and third group (green) from month 13 to 18. Implementation seminars will be held at months 0, 6 and 12, and midway evaluation at months 3, 9 and 15. Data will be collected at baseline (month 0), after the first intervention period (month 6–7), after the second intervention period (month 12–13), after the third intervention period (month 18–19) and at the end of the study at 24 months. **b** Schedule of enrollment, interventions and assessments over the study period

Data from all 315 dyads will be assessed every 6 months from baseline to the end of study period after 24 months, death or permanent residency in a nursing home—in total, five waves of data collection. The stepped-wedge randomized control design [61] implies that all participants will receive the 6-month intervention program during the study period, for which the *timing* of the intervention is determined by the randomization (Fig. 2). The control group constitutes the dyads waiting for the intervention at a given time during the study; this group will have access to health care and receive treatment as usual. Criteria for discontinuing the intervention or participation are requested from participants to withdraw from the trial. The trial’s user-oriented approach, aiming at minimizing the participant burden associated with follow-up visits, in addition to flexibility in scheduling of the visits are sought to promote retention and prevent loss to follow-up over the trial. No distinct adverse events are expected before the start of the trial or during the trial, while possible adverse events related to the change in prescribed medication during the general practitioner’s medication review might occur. If so, they will be reported by the coordinators to the researchers, either immediately or at their regular follow-up every 2 weeks (physical meeting, by phone or by e-mail), in addition to feedback from the coordinator to the general practitioner. A statistician will randomly allocate the order of the intervention using block randomization; the dyads are randomized in clusters within each geographical location. The random sequence will be generated using a computerized random number generator undertaken for all three municipalities after the inclusion and baseline assessments are completed for all participants. Research assistants, researchers conducting the analyses and other study personal conducting data collection will be blind to the randomization order and to the implementation process of the intervention. Participants will not be informed of the intervention and implementation strategy to secure blinding until they are allocated to their coordinator during the intervention period. From this point of time, they become unblinded. Given the practice change of the intervention, the municipality homecare services will be aware when their cluster enters the intervention period.

When developing a pathway for dementia care, incorporating experiences and perspectives from the PWDs and their caregivers is fundamental. In line with the INVOLVE framework [62], this trial is developed through user involvement from the conception of the idea, via design through the implementation phase. At the structural level, user involvement is secured via collaboration with the head of research at the Norwegian Health Associations [51], participating in the Steering committee, and locally grounded by dementia coordinators in the municipalities. At the individual level, the Centre for Elderly and Nursing Home Studies (SEFAS), responsible for conducting the trial, employs a user-representative as a co-researcher in a 10% position, who participates in the study’s advisory board and working group. The mixed-method design [63] encompasses the integration of data from quantitative assessment of validated outcomes with material from qualitative interviews and participant observation. Utilizing an exploratory hermeneutic design [64], in-depth and focus group interviews with PWDs (*n* = 15), caregivers (*n* = 15), municipality health-care staff (*n* = 20), general practitioners (*n* = 10), volunteers (*n* = 18) and volunteer coordinators (*n* = 6) will be conducted. To evaluate the acceptability and feasibility of the communication platform, interviews with caregivers and care staff will be made, as well as real-life observations form use among PWDs and caregivers.

### Outcome measures

Table 2 presents the primary and secondary outcomes according to domain, specific measurement, metric, method of aggregation and time points. The primary outcome of the LIVE@Home.Path trial is formal and informal resource utilization, measured by the RUD instrument [65, 66] and the RSS [67] (Table 2). As such, we consider overall resource utilization as more than the time required to care for the PWD; it also encompasses how burdensome the task is experienced by the caregiver. The informal care time use is measured in hours/month [65, 68], in addition to numbers of contacts with the health-care system and use of medications. The RUD is a standardized and widely used instrument assessing dementia care, proven useful across different care systems and countries and in both clinical trials and observational studies [65, 66]. Caregivers stress will be assessed by the RSS, a self-report instrument covering three dimensions of “emotional distress”, “social distress” and “negative feelings”. It is regarded as a useful instrument to stratify careers according to the risk of psychiatric morbidity [69, 70].

**Table 2:** Primary and secondary outcomes in the LIVE@Home.Path trial

Domain: name of tool	Specific measurement: what the tool measures	Characteristics of tool	Metric	Method of aggregation	Time points
Primary outcome					
Resource Utilization in Dementia (RUD) (65, 66, 68)	Resource utilization in dementia care	Self-reported formal and informal care time use in hours/30 days on activities of daily living (e.g. feeding, dressing, bathing) and supervision (e.g. wandering, preventing dangerous situations) Assess number of contacts with health-care professionals for both PWD and caregivers in the last 30 days, and use of medications High number of hours of direct care time and numerous contacts with health-care professionals indicates high overall resource use in dementia care	Change in hours/30 days	Mean	Mean difference in hours/30 days over the 6-month intervention period summarized for the three intervention groups compared to mean difference in hours/30 days summarized for the control groups^a^ Mean difference in hours/30 days over the follow-up period in 6-month intervals stratified by time from end of intervention^b^
Relative Stress Scale (RSS) (69, 70)	Caregiver distress	15 items for self-report of three subgroups of distress: “emotional distress”, “social distress” and “negative feelings” Each item ranging from 0 to 4 High score indicates high burden and psychiatric morbidity	Change in total score	Mean	Mean difference in score over the 6-month intervention period summarized for the three intervention groups compared to mean difference in score summarized for the control groups^a^ Mean difference in score over the follow-up period in 6-month intervals stratified by time from end of intervention^b^
Secondary outcomes					
European Quality of Life—5 Dimensions—5 Levels (EQ-5D-5L) (72)	Generic quality of life	Evaluates generic self-reported health-related quality of life in relation to resource use Five items regarding mobility, self-care, activities, pain/discomfort and anxiety/depression scored on a five-level scale Scores are converted to a single summary index number	Change in total score	Mean	Mean difference in score over the 6-month intervention period summarized for the three intervention groups compared to mean difference in score summarized for the control groups^a^ Mean difference in score over the follow-up period in 6-month intervals stratified by time from end of intervention^b^
EQ-5D-VAS scale (73)	Quality of Life-VAS scale	One-point measure of generic self-reported health-related quality of life rated on a visual analog scale from 0 to 100, high score indicates good quality of life	Change in score	Mean	Mean difference in score over the 6-month intervention period summarized for the three intervention groups compared to mean difference in score summarized for the control groups^a^ Mean difference in score over the follow-up period in 6-month intervals stratified by time from end of intervention^b^
Quality of Life in Alzheimer’s disease scale (QoL-AD) (71)	Quality of life in Alzheimer’s dementia	Disease-specific self-reported quality of life measure assessing13 items each ranging from 1 to 4 High score indicates high quality of life	Change in total score	Mean	Mean difference in score over the 6-month intervention period summarized for the three intervention groups compared to mean difference in score summarized for the control groups^a^ Mean difference in score over the follow-up period in 6-month intervals stratified by time from end of intervention^b^
Neuropsychiatric Inventory, 12-item version with caregiver distress (NPI-12) (74)	Neuropsychiatric symptoms in dementia	Proxy-rated presence, severity and caregiver distress of 12 items assessing depression, anxiety, psychosis and motor disturbances Range 0–144, high score indicates frequent and severe symptoms The distress scale assess caregiver distress associated with each neuropsychiatric symptom, range 0–60, high score indicate distressing symptoms	Change in total score and change in score for each item	Mean, and proportion above clinical significant score	Mean difference in total and item specific score over the 6-month intervention period summarized for the three intervention groups compared to mean difference in score summarized for the control groups^a^ Mean difference in total and item specific score over the follow-up period in 6-month intervals stratified by time from end of intervention^b^
Cohen-Mansfield Agitation Inventory (CMAI) (75, 76)	Agitation in dementia	29 items rated from 1 to 7 for proxy assessment frequency of agitated behavior Range 29–203, high score indicates frequent agitation	Change in total score	Mean and proportion above clinical significant score	Mean difference in score over the 6-month intervention period summarized for the three intervention groups compared to mean difference in score summarized for the control groups^a^ Mean difference in score over the follow-up period in 6-month intervals stratified by time from end of intervention^b^
Cornell Scale for Depression in Dementia (CSDD) (77)	Depression in dementia	19 items rated from 0 to 2 for proxy assessment of depressive symptoms in dementia Range 0–38 Score ≥ 8 indicates depression; ≥ 12 indicates moderate–severe depression	Change in total score	Mean and proportion above clinical significant score	Mean difference in score over the 6-month intervention period summarized for the three intervention groups compared to mean difference in score summarized for the control groups^a^ Mean difference in score over the follow-up period in 6-month intervals stratified by time from end of intervention^b^
Geriatric Depression Scale (GDS) (78)	Depression in old age	30 items rated 0 or 1, for proxy assessment of depressive symptoms in the elderly population High score indicates high symptom load	Change in total score	Mean	Mean difference in score over the 6-month intervention period summarized for the three intervention groups compared to mean difference in score summarized for the control groups^a^ Mean difference in score over the follow-up period in 6-month intervals stratified by time from end of intervention^b^
Activities of Daily Living, Instrumental (I-ADL) (76)	Functional level for instrumental activities	Eight items for proxy assessment of use of telephone, shopping, economy, public transport and household Range 8–31, high score indicates poor functioning	Change in total score	Mean	Mean difference in score over the 6-month intervention period summarized for the three intervention groups compared to mean difference in score summarized for the control groups^a^ Mean difference in score over the follow-up period in 6-month intervals stratified by time from end of intervention^b^
Activities of Daily Living, Personal (P-ADL) (79)	Functional level for personal activities	Six items rated 1–5 for proxy assessment of personal activities such as toileting, grooming, dressing, transfer and eating Range 6–30, high score indicates poor functioning	Change in total score	Mean	Mean difference in score over the 6-month intervention period summarized for the three intervention groups compared to mean difference in score summarized for the control groups^a^ Mean difference in score over the follow-up period in 6-month intervals stratified by time from end of intervention^b^
General Medical Health Rating Scale (GMRH) (85)	Medical comorbidity in dementia	4-point Likert scale assessing presence and severity of medical conditions, scored by the interviewer High score indicates high comorbidity burden	Ratings on the Likert scale transformed to numeric scale to estimate change in total score	Mean	Mean difference in score over the 6-month intervention period summarized for the three intervention groups compared to mean difference in score summarized for the control groups^a^ Mean difference in score over the follow-up period in 6-month intervals stratified by time from end of intervention^b^
Mobilization–Observation–Behavior–Intensity Dementia Pain Scale (MOBID-2) (80–84)	Pain in dementia	10 items rated 0–10 for proxy-rated assessment of pain related to the muscle–skeletal system and pain that might be related to internal organs, head and skin High score indicates frequent and severe pain	Change in overall score and change in score for each item	Mean	Mean difference in score over the 6-month intervention period summarized for the three intervention groups compared to mean difference in score summarized for the control groups^a^ Mean difference in score over the follow-up period in 6-month intervals stratified by time from end of intervention^b^
Clinical Global Impression of Change (CGIC) (86)	Clinical meaningful change	Quantifies and tracks patient progress and treatment response on a scale from 1 to 7, scored by the interviewer High score indicates worsening	Change in overall score	Mean and proportion with worsening, no change and improvement	Mean difference in score over the 6-month intervention period summarized for the three intervention groups compared to mean difference in score summarized for the control groups^a^ Mean difference in score over the follow-up period in 6-month intervals stratified by time from end of intervention^b^

All assessment will be made by research personal or affiliated staff in the municipalities during home visits with the person with disability (PWD) and the caregiver

^a^Intervention groups: group 1 (red), t1–t2; group 2 (yellow), t2–t3; group 3 (green), t3–t4. Control groups: (t1–t2 + t2–t3) (see Fig. 2a)

^b^Group 1 (red): three 6-month periods, t2–t3, t3–t4 and t4–t5. Group 2 (yellow): two 6-month periods, t3–t4 and t4–t5. Group 3 (green): one 6-month period, t4–t5 (see Fig. 2a)

The secondary outcomes presented in Table 2 include measures of QoL, psychiatric symptom load, ADL, comorbidity and pain as well as measure of goal achievements. The QoL for both the PWD and the caregiver will be measured by self-report using the Quality of Life in Alzheimer’s disease scale (QoL-AD) [71] and the generic quality of life measure EQ-5D-5L [72], including the EQ-5D-VAS scale [73]. Additionally, QoL for the PWD will be assessed by proxy by the caregiver with the QoL-AD [71]. Psychiatric symptoms for the PWD will be proxy rated by the caregiver using the Neuropsychiatric Inventory Questionnaire (NPI-12) [74], the Cohen-Mansfield Agitation Inventory (CMAI) [75, 76] and the Cornell Scale for Depression in Dementia (CSDD) [77], while caregiver psychiatric symptoms will be self-reported using the Geriatric Depression Scale (GDS) [78] in addition to the RSS [67]. Data on ADL for the PWD will be proxy rated by the caregiver utilizing instrumental (I-ADL) and personal (P-ADL) measures [79]. Data on pain will be obtained by self-report from the PWD using the MOBID-2 Pain Scale [80–84] and the level of comorbidity will be evaluated by the interviewer according to the General Medical Health Rating Scale (GMRH) [85]. The Clinical Global Impression of Change Scale (CGIC) will be assessed after the intervention to quantify and track patient progress and treatment response [86]. In addition to the instruments presented in Table 2, other outcome measures include the number of adverse events (falls, disappearances outdoors, fire hazard), use of assistive technology (number of technical aids, cognitive intervention devices and assisted living systems), involvement of volunteers (number of participants with contact with a volunteer, number of hours spent with a volunteer), number of medications used (both regular and on demand) and participation in educational programs for the PWD and the caregiver. These outcome measures will be described as the mean change in sum of events (number devices, hours, medications, educational programs) over the intervention period compared to controls (as outlined in Table 2).

### Quantitative data quality and collection

Prior to inclusion and baseline data collection, a 1-day seminar will be arranged for the study personal to secure training in the use of tablets and scoring of relevant psychometric scales. A study manual has been developed to guide data collectors during their visits to secure standardized reporting. Close to 24 h/day, telephone and mail support will be offered by the research team during times of data collection. Researchers and municipal study personal will collect data at baseline as well as 6, 12, 18 and 24-month follow-up. The municipalities will receive 5000 NOK per enrolled dyad to compensate for extra administrative work. At baseline, demographic data such as year of birth, gender, marital status, housing characteristics, education and employment will be collected, as well as data on the dementia syndrome, including the current score on the Mini-Mental State Examination, Norwegian Version (MMSE-NR3) [87, 88], Functional Assessment Staging Test (FAST) [89] and The Informant Questionnaire on Cognitive Decline in the Elderly (IQCODE) [90, 91]. The MMSE-NR3 [88] will be assessed every 12 months during the trial.

### Plan for quantitative data analyses

Intention-to-treat analyses will be performed accounting for municipality as a random effect in mixed-effect models and the generalized estimating equation (GEE) with nonlinear effect comparing the intervention groups to controls. Repeated observations within persons will be accounted for with a correlation matrix. All secondary outcomes will be adjusted for multiple comparisons using the Hochberg method [92]. Given the potentially informative censoring due to dropout, institutionalization and death, we will jointly model the primary outcome and attrition through a shared person-specific random intercept. Missing data will be handled using multiple imputations by chained equations (MICE).

### Ethical approval

The study was approved in May 2019 by the Regional Committee for Medical and Health Research Ethics, North Norway (2019/385) and West Norway (2017/1519) (the pilot), and registered at ClinicalTrials.gov (NCT04043364). Assessment and utilization of personal data from the dyads as well as from volunteers and volunteer coordinators from nonprofit organizations are approved by the Norwegian Centre for Research Data (NSD) (ref. 514093). After verbal and written information, spoken and written informed consent was obtained in direct conversation with the caregiver and the PWD, if capable of providing consent for participation. If not, the next of kin or a legal advocate provided consent based on their determination on whether the PWD, when they were able, would have agreed to participate in the trial.

## Discussion

Compared to care as usual, we expect the LIVE@Home.Path trial to innovate the clinical pathway in dementia care, facilitating cost-effective, feasible and independent living at home through Learning, Innovation, Volunteering and Empowerment.

### Framework for sustainable ethic innovation in dementia research

Participation in research is based on affirmative, unambiguous, informed and specific consent [93]. Persons with cognitive impairment will often not be able to provide such a comprehensive consent or understand the scope and consequences of data assessment. Local legislation for obtaining ethical permission in studies varies substantially between European countries [94]. In Norway, the next of kin or a legal advocate can provide consent based on their determination of whether the person, when they were able, would have agreed to participate in the trial [23]. These principles for obtaining informed consent were applied in the LIVE@Home.Path trial. From 2018, the European Union-wide law on data protection, the General Data Protection Regulation (GDPR), represents a significant step toward protection of participants in research [95]. In particular, Article 6 protects PWDs and their relatives from being coerced to consent without awareness of how their data will be used [96, 97]. When assessing sensitive data such as mental health, Article 35 requires a Data Protection Impact Assessment (DPIA), a formal process systematically analyzing, identifying and minimizing the data protection risks of a project. We developed a DPIA (ePhorte UiB: 2019/5569) for the LIVE@Home.Path trial in collaboration with the Data Protection Official at the University of Bergen, encouraging us to again evaluate which data to assess, as well as focus on safe data management. Nonetheless, we anticipate the participation in the LIVE@Home.Path trial to be less burdensome relative to, for example, RCTs on effect of medications, due to the user-oriented approach emphasizing the investigation of the perspective “What matters to you?”

**Fig. 3:**
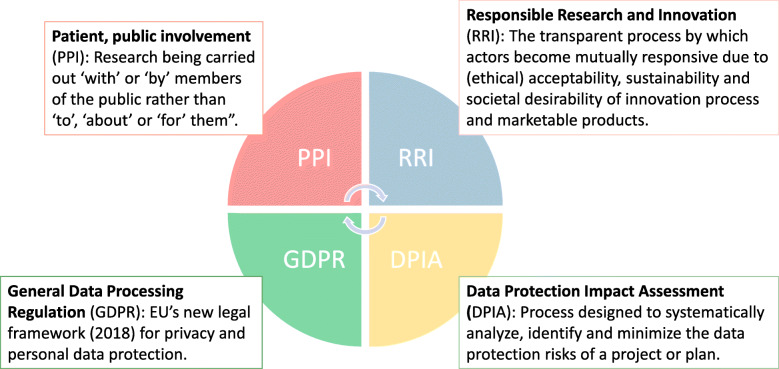
Framework for sustainable ethic innovation in dementia research

Stakeholders and research funders increasingly require patient and public involvement (PPI) at all stages of research from design, implementation and dissemination of results, shifting focus from research “about” or “for” to research “with” or “by” someone [98, 99]. Our user-representative has provided feedback on a close to weekly basis through participation in the working group and advisory board of the trial. A related principle, Responsible Research and Innovation (RRI), is defined as a transparent, interactive process making societal actors and innovators mutually responsive to each other, and encouraging them to set up a critical perspective when evaluating the innovation and marketability of products [100, 101]. Taken together, these components constitute a framework for sustainable ethic innovation in dementia research (Fig. 3), a model that easily can be applied when designing and conducting research on other vulnerable patient groups.

### Methodological considerations

A stepped-wedge randomized controlled trial design is recommended for evaluation of a multicomponent intervention in health-care services as it provides a number of practical and scientific benefits compared to an ordinary RCT [61]. It is increasingly used in effectiveness studies in the geriatric field [102, 103]. Most importantly, the design allows for providing the intervention to all participants, overcoming ethical and logistical challenges arising from withholding the intervention. This design is, however, more vulnerable to temporal external changes, as more participants are exposed to the intervention toward the end of the study than in earlier stages. If the LIVE intervention fails to prove an effect on resource utilization, we will examine whether this is due to a lack of proper implementation. Thus, if the implementation process is satisfactory, it may suggest that the LIVE components were not tailored to be sufficiently cost-effective if no effects on primary outcome measures are found. An alternative interpretation is that the intervention may not be cost-effective even if primary outcomes change significantly, as resource use by the intervention is more time consuming and/or expensive than the alternative.

### Practical pitfalls and obstacles

Some challenges have emerged during the start of the trial. First, it is demanding to include the estimated number of participants, and, additionally, to keep the number of dropouts low due to the progression of the disease. We should have established closer collaborations with the geriatric specialist health-care services, as we experienced that patients recruited from geriatric outpatient clinics were in the most optimal disease stage for this trial. To increase recruitment, we prolonged the inclusion period to 31 December 2019 and expanded the inclusion criteria to age ≥ 64 years and MMSE range 15–27, while the SEFAS researchers, journalist and co-researcher with user experience continuously work on positive media coverage. Second, data collection from home-dwelling persons in three distinct municipalities is resource and logistically demanding. Third, being selected as a pilot for the data collection software has been challenging, as the file format initially generated handled missing data in a way that was not compatible with our statistical programs. Finally, the participants have so far been recruited in various ways, from home-care services in the municipality and memory clinics at hospitals, to self-referrals after advertisements in the general media. This implies that the dyads included in our trial represent a heterogeneic group of home-dwelling people with dementia.

In conclusion, we expect the implementation of LIVE to lead to a pathway for dementia treatment and care that is cost-effective, feasible and supports independent living, at home.

## Trial status

A total of 428 dyads had been screened for participation from 20 May 2019, of which 279 were included in the trial. By January 2020, when recruitment ended, 31 dyads had dropped out. Mainly due to a more rapid inclusion process than anticipated, this protocol was submitted after the end of the recruitment period but in due time before the last visit for data collection. At the time of resubmission in May 2020, the COVID-19 pandemic had profoundly impacted the Norwegian health-care system, including services in the municipal sector, challenging the implementation of the intervention in group 2. Newsletters with status, possible modifications and upcoming events will be sent by e-mail to the site leaders and coordinators every 2–3 months. Final protocol version number 5 will be prepared by 1 June 2019.

## Supplementary information

**Additional file 1.** Implementation seminar for the LIVE@Home.Path trial.

## Data Availability

Data sharing is not applicable to this article as no datasets were generated or analyzed during the current study. The public will not receive full access to the complete protocol, dataset and statistical procedures; however, this information can be made available to other researchers upon request.

## Abbreviations

ACP: Advanced care planning
ADL: Activities of daily living
CGIC: Clinical Global Impression of Change
CMAI: Cohen-Mansfield Agitation Inventory
CSDD: Cornell Scale for Depression in Dementia
DPIA: Data Protection Impact Assessment
EQ-5D-5L: European Quality of Life—5 Dimensions—5 Levels
FAST: Functional Assessment Staging Test
FTE: Full-time equivalents
GDPR: General Data Protection Regulation
GDS: Geriatric Depression Scale
GMRH: General Medical Health Rating Scale
I-ADL: Activities of Daily Living, Instrumental
ICT: Information and communication technology
LIVE: Learning, Innovation, Volunteering, Empowerment
MOBID-2: Mobilization–Observation–Behavior–Intensity Dementia Pain Scale
MMSE: Mini-Mental State Examination
NPI-12: The Neuropsychiatric Inventory Questionnaire
P-ADL: Activities of Daily Living, Personal
PPI: Patient and public involvement
PWD: Person with dementia
QoL: Quality of life
QoL-AD: Quality of Life in Alzheimer’s disease scale
RCT: Randomized controlled trial
RRI: Responsible Research and Innovation
RSS: Relative Stress Scale
RUD: Resource Utilization in Dementia
SEFAS: Centre for Elderly and Nursing Home Medicine
