# Supplementary material for: LIVE@Home.Path—innovating the clinical pathway for home-dwelling people with dementia and their caregivers: study protocol for a mixed-method, stepped-wedge, randomized controlled trial
Source: Trials. 2020 Jun 9;21:510. doi: 10.1186/s13063-020-04414-y (PMC7281688; doi:10.1186/s13063-020-04414-y)
Supplement: Supplementary file 1 — Additional file 1. Implementation seminar for the LIVE@Home.Path trial. [file 13063_2020_4414_MOESM1_ESM.docx]

**Implementation seminar for the** [**LIVE@Home.Path**](mailto:LIVE@Home.Path) **trial**

**When:** 2 days in August 2019

**Where:** Municipal conference center

**Who:** Principal investigator, research team and local coordinators

**Day 1**

09.00-09.10 Welcome by local contact person

09.10-09.15 About LIVE@Home.Path trial

09.15-10.15 The coordinator in [LIVE@Home.Path](mailto:LIVE@Home.Path) trial

10.15-11.00 Learning in dementia

11.00-11.15 Break

11.00-12.00 Group work and general discussion

12.00-12.30 Lunch

12.30-13.00 Innovation in dementia: welfare technology

13.00-13.30 Empowerment in dementia

13.30-13.45 Break

13.45-14.30 Group work and general discussion

14.30-15.00 The checklist for implementation in the [LIVE@Home.Path](mailto:LIVE@Home.Path) trial

**Day 2**

09.00-10.00 Case presentation and general discussion

10.00-10.15 The medication review in the LIVE@Home.Path

10.15-10.30 Break

10.30-11.30 Volunteering in dementia care

11.30-12.00 Group work and general discussion

12.00-12.30 Lunch

12.30-13.15 Case presentation and general discussion

13.15-14.00 Case presentation and general discussion

14.00-14.15 Break

**
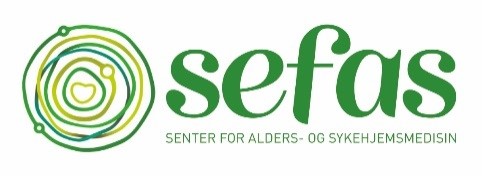

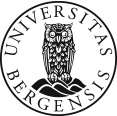
**14.15-15.00 Closing remarks and good luck!
